# Repeatability of the amplitude of accommodation measured by a new generation autorefractor

**DOI:** 10.1371/journal.pone.0224733

**Published:** 2020-01-27

**Authors:** Chang-Chi Weng, De-Kuang Hwang, Catherine Jui-Ling Liu

**Affiliations:** 1 Department of Ophthalmology, Taipei Veterans General Hospital, Taipei, Taiwan; 2 Faculty of Medicine, National Yang-Ming University School of Medicine, Taipei, Taiwan; Faculty of Medicine, Cairo University, EGYPT

## Abstract

**Significance:**

This is the first study to validate the repeatability of objective measurements of amplitude of accommodation (AA) using the TONOREF III (NIDEK Co., Ltd., Japan), which can measure the AA in 30 seconds.

**Purpose:**

To evaluate the repeatability of objective measurements of AA using the TONOREF III and explored the association between objectively measured AA and factors, including age, sex, spherical equivalent, baseline pupil size and pupil size change during accommodation.

**Methods:**

This cross-sectional study recruited 35 healthy subjects aged 26 to 52 years. The Bland-Altman method and intraclass correlation coefficients (ICCs) were used to assess the repeatability of TONOREF III measurements. The relationships between AA and age, sex, baseline pupil size, changes in pupil size (ΔPS) during accommodation were analyzed using linear regression for univariate and multivariate analysis.

**Results:**

The mean difference in AA (ΔAA) between two sessions of TONOREF III measurements was 0.23 D (95% CI: -1.07 to +1.53 D), while no significant correlation was found between the mean and ΔAA (*p* = .14). The ICCs of the TONOREF III was 0.96. Age, sex, and ΔPS during accommodation were significant factors affecting the AA in multivariate analysis.

**Conclusions:**

The repeatability of objective AA measurements using the TONOREF III was good. Measuring AA using the TONOREF^TM^ III in clinical practice is feasible.

## Introduction

Accommodation is adjustment in the refractive power of the crystalline lens so that the images of objects can be brought into focus on the retina over a range of distances. The amplitude of accommodation (AA) is the maximal potential increase in optical power that an eye can achieve in adjusting its focus. The change in refraction of the eye measured by an autorefractor while the subject accommodates from a distant to near target represents an objective measurement of accommodation. Several studies measuring the AA objectively using an open-field autorefractor have demonstrated a lag in accommodation that is the amount by which the accommodative response is less than the dioptric stimulus to accommodation [1–5]. The AA measured subjectively is at best near vision capacity rather than the actual refractive changes of the eye, whereas objective measurements could represent actual changes in the optic power of the eye [1–4].

In daily clinical practice, sometimes there are young adults and middle-aged patients complaining about eye strain or blurred vision when seeing near objects. It is worthwhile to identify if they had decreased accommodation in their ages and the actual diopter of the AA using objective measurements. Because diabetes mellitus, Down syndrome or drugs such as topiramate may lead to early decreased accommodation [6–9], timely diagnosis and intervention of these underlying causes may be helpful to patients. For new therapy proclaiming that it can restore accommodation for patients with early presbyopia [10], it is also helpful to validate the effect using objective measurements. In addition, the use of electronic devices may be associated with decreased accommodation and its association with aesthenopic symptoms is worth further investigation with objective measurement [11].

The NIDEK TONOREF III (NIDEK Co., Ltd., Japan) has a built-in auto refractometer, auto keratometer, non-contact tonometer, and non-contact pachymeter. It has the advantage of taking up less space and time-saving for measuring the refraction, intraocular pressure, central corneal thickness and AA using the same machine. Different from the Shin-Nippon SRW-5000 or Grand-Seiko WR-5100K open-field autorefractors using an external target, it dynamically and simultaneously measures the AA as well as the changes in pupil size in 30 seconds using an internally virtual object. The mechanism of measuring refraction and accommodation is identical to the NIDEK Auto Ref / Keratometer (ARK-1s / 1a). In brief, objective measurements of accommodation are obtained dynamically by measuring the dioptric change of the subject while the examinee is focusing on a virtual object which moves from distance to near. To the best of our knowledge, no previous study has reported the repeatability of AA measurements using the TONOREF III.

The present study aimed to evaluate the repeatability of objective measurements of AA using the TONOREF III. Since age is a known factor affecting the accommodation [2], we investigated the associations between the measured AA and age to verify clinical feasibility of the TONOREF III. Because previous studies argued that sex and refractive errors were factors affecting AA and the results remained controversial [12–18]. It is worthwhile to evaluate these factors. Besides, since TONOREF III can measure the pupil size and refractive change during accommodation at the same time, it is interesting to explore the association between pupil size and AA. As a result, factors including sex, refractive errors, baseline pupil size and its change during accommodation were also investigated.

## Methods

This cross-sectional study followed the tenets of the Declaration of Helsinki and was approved by the Institutional Review Board of Taipei Veterans General Hospital, Taipei, Taiwan (VGH IRB number: 201803002BC). Written informed consent was obtained from all participants.

Adult subjects were recruited from March 2018 to July 2018. The inclusion criteria were as follows: best corrected Snellen visual acuity better or equal to 0.8 (logMAR 0.1), no strabismus, no history of ocular trauma, no ocular diseases except refractive errors which was confirmed using slit-lamp biomicroscopy by one ophthalmologist, no diabetes mellitus or taking drugs which may have affected the subject’s accommodation such as topiramate, topical atropine, pilocarpine, and tropicamide.

Refractive errors (vertex distance = 12mm) were measured using the TONOREFTM III. All participants were wearing glasses with full correction for distance (6 meter) visual acuity measurement. The dominant eye of each subject was determined according to the Miles test. The reference position of the internal target is the sphere value of the measured refractive errors (vertex distance = 12mm) and the initial position is sphere value + 0.5D. The subjects viewed the internal target monocularly without contact lens or spectacles in the TONOREF III which moved from distance (initial position) to near. While the target moved from the initial position, successive measurements of refraction and pupil size were taken at the same time for a maximum of 30 seconds. The measuring rate depended on the subject’s response (refractive change). If the instrument detected no refractive change for continuous 6 seconds, measurement finished. After the measurements were complete, the AA was calculated as the difference between the maximum and minimum refraction value (vertex distance = 12mm). The pupil size change was defined as the difference between the baseline and minimum pupil size during accommodation. For each subject, the objective AA was measured in the dominant eye twice under fixed ambient light using the TONOREF III by the same operator with a 10-minute interval. The subjects were asked not to perform near visual tasks such as using cellphones, reading, or writing during the 10-minute interval because near visual tasks may affect the result of measured accommodation [11]. If the subjects had never been examined using a TONOREF^TM^ III before, a pre-test was performed to allow them to become familiar with the whole procedure to minimize the effects of a learning curve.

Data were analyzed using Statistical Package of Social Sciences software (version 20.0; SPSS, Inc., Chicago, IL, USA). The Bland-Altman method and intraclass correlation coefficients were used to assess the repeatability of TONOREF^TM^ III measurements [19, 20]. The relationships between the AA in the dominant eye and age, sex, spherical equivalent, baseline pupil size, changes in pupil size during the measurements, were analyzed using linear regression for univariate and multivariate analysis adjusting for age, sex, baseline pupil size, changes in pupil size during accommodation, spherical equivalent. Mallows' Cp was used for model selection in multivariate analysis. The significance level was considered as *p* < .05 in all tests.

## Results

Thirty-five subjects were enrolled, of whom 27 were female (77.1%) and 8 were male (22.9%). The mean age (±SD) was 35.9 (±7.4) years (range: 26–52 years), and there was no significant difference in mean age between the males and females (*p* = .11). The average spherical equivalent was -5.24 (±3.16) D (ranging from plano to -10.63 D). Baseline pupil size, changes in pupil size during accommodation, AA measured by the TONOREF III, and difference between two sessions of measurements are shown in Table 1.

**Table 1:** Baseline characteristics of the participants (n = 35).

Variables	Mean	SD
Age (year)	35.89	7.35
SE (D)	-5.24	3.16
Baseline pupil size (mm)	5.68	0.87
Changes in pupil size during accommodation (mm)	1.22	0.62
AA measured by the autorefractor (D)	3.29	2.35
ΔAA between two sessions of measurements (D)	0.23	0.66

AA, amplitude of accommodation; ΔAA, the difference in amplitude of accommodation; D, diopter; mm, millimeter; SE, spherical equivalent; SD, standard deviation.

**Fig 1:**
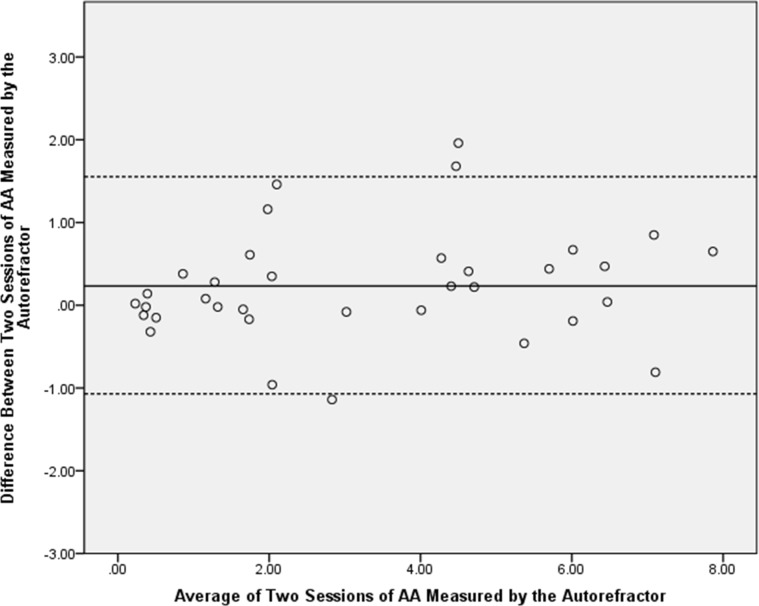
The Bland-Altman plots of repeatability for accommodative amplitude (AA) measurement. Repeatability of AA measured by the autorefractor. Mean difference, 0.23 D; 95% confidence limit, -1.07 to +1.53 D.

The result of the Bland-Altman method for the repeatability of TONOREF III is shown in Fig 1. The mean difference and 95% limits of agreement were small, and there was no tendency for the difference to increase with mean AA (*p* = .14). The intraclass correlation coefficients for the measurements of AA by the TONOREF^TM^ III was 0.96.

Factors affecting the amplitude of accommodation are shown in Table 2. Age, sex, and changes in pupil size during accommodation were significant factors affecting the AA in univariate analysis and multivariate analysis as well. The Mallow’s Cp of this multivariate model was 5.00. The factors affecting the AA in the female subjects are shown in S1 Table and the results remained similar.

**Table 2:** Factors affecting the amplitude of accommodation.

Factors	Univariate analysis		Multivariate analysis*	
	coefficient	*P* value	coefficient	*P* value
Age	-0.25	< .0001	-0.17	< .0001
Sex**	2.63	.004	1.06	.028
Baseline PS	-0.29	.546	-0.38	.08
ΔPS during Accommodation (mean)	2.92	< .0001	1.49	< .0001
Spherical equivalent (mean)	-0.21	.106		

PS, pupil size. ΔPS, changes in pupil size.

*Multivariate analysis adjusted for age, sex, changes in pupil size during accommodation.

**Coefficient > 0 means that male has higher amplitude of accommodation.

## Discussion

The value of measured AA varies using different measurements and there is no consensus about which one is the optimal method. Although Hoffstetter formula is commonly used for AA estimation in clinical practice, it is shown to have higher predicted AA than objective measurements in previous studies [2, 3]. It is more appropriate to compare the AA in this study with other objective measurements. Because our findings showed similar trends with the study results of Andersone et al. [1], it is likely that the value of AA measured by TONOREF III is valid representation of objective AA measurement.

In cases of good repeatability of measurements, the mean difference would be close to 0, the 95% limits of agreement would be small, and the difference in AA would not change with its mean in Bland-Altman plots [19]. An intraclass correlation coefficient greater than 0.9 means excellent repeatability [21]. Therefore, this study demonstrated that the TONOREF^TM^ III had good repeatability of the measurements of AA, since the mean difference and 95% limits of agreement were small, and there was no tendency for the difference to increase with mean AA (*p* = .14). Since the distribution of age and myopic refraction was wide among the participants, we had the opportunity to demonstrate that the repeatability remained good in participants with high myopia or at middle age. This is important for clinical application of TONOREF^TM^ III when it comes to evaluation of AA, because patients with accommodative insufficiency usually are in or near middle age and there is high prevalence of myopia in East Asia [22]. Previous studies have demonstrated good repeatability and accuracy of other objective measurements of accommodation, such as the WR-5100K autorefractor, iTrace wavefront aberrometer, and Hartinger coincidence refractometer[2, 5, 23]. However, to the best of our knowledge, no prior study has validated the repeatability of the TONOREF^TM^ III.

In this study, factors affecting the AA were age, sex and changes in pupil size during accommodation. Since age is a known factor affecting the accommodation [1, 2], this study indirectly verified the credibility of TONOREF III in measuring the AA. Besides, we found that sex and changes in pupil size during accommodation were correlated with the AA. Sex remained a controversial factor affecting the accommodation because the association between sex and AA varied in different studies [15–18]. In the current study, the female subjects had a lower AA as measured by the TONOREF III than the male subjects in multivariate analysis. The reason why sex is a significant factor affecting accommodation remains unknown. Since the AA used for analysis in the current study was objectively measured and factors which may influence the AA were adjusted in multivariate analysis, our findings regarding sex seem to be plausible. To the best of our knowledge, no previous study explored the association between pupil size change during accommodation and AA. Since accommodation and the pupillary near response as in miosis share a common neural controller which integrates stimuli for accommodation such as blurring [24], we speculate that more prominent miosis is associated with greater sensitivity to stimuli, and that the AA is associated with the sensitivity to stimuli. However, studies using this machine with larger sample size were needed to confirm these findings.

The importance of this study is that after validating the repeatability and accuracy of TONOREF III, it could be applied to the daily clinical practice and research. With the advantages of time-saving and space-efficient design, TONOREF III could facilitate the busy clinical practice. In addition to identifying patients with early accommodation decrease and timely diagnosis of underlying disease such as diabetes mellitus [6], TONOREF III can be used to evaluate the effect of new therapy proclaiming restoring accommodation. Furthermore, studies focusing on factors affecting objective accommodation can be performed using TONOREF III.

This study was limited by its small sample size which may have underestimated the significance of the correlations between the evaluated factors and AA. However, the finding that the male subjects had a significantly higher AA than the female subjects suggests that the sample size may be reasonable for a study of this kind. Second, since most participants had myopia in this study, further studies investigating the repeatability and associated factors in hyperopic eyes may be needed. Third, since the participants were adults without ocular disease in this study, the repeatability of TONOREF III in children or patients with ocular disease who may have decreased AA needs further evaluation in future studies.

In conclusion, the TONOREF III exhibited good repeatability of AA measurements. Our results indicated that AA had significant association with age, sex, and changes in pupil size during accommodation.

## Supporting information

S1 Table Factors affecting the amplitude of accommodation in female participants (n = 27). PS, pupil size. ΔPS, changes in pupil size. *Multivariate analysis adjusted for age, baseline pupil size, changes in pupil size during accommodation, spherical equivalent.
(PDF)

S1 File Raw data for replicating this study findings. (XLSX)
