# Supplementary material for: Repeatability of the amplitude of accommodation measured by a new generation autorefractor
Source: PLoS One. 2020 Jan 27;15(1):e0224733. doi: 10.1371/journal.pone.0224733 (PMC6984687; doi:10.1371/journal.pone.0224733)
Supplement: S1 Table — PS, pupil size. ΔPS, changes in pupil size. *Multivariate analysis adjusted for age, baseline pupil size, changes in pupil size during accommodation, spherical equivalent. (PDF) [file pone.0224733.s001.pdf]

S1 Table. Factors affecting the amplitude of accommodation in the females (n=27)

Factors	Univariate analysis		Multivariate analysis*	
	coefficient	*P* value	coefficient	*P* value
Age	-0.23	<.0001	-0.16	<.0001
Baseline PS	-0.47	0.34	-0.20	0.45
ΔPS during accommodation (mean)	2.77	<.0001	1.64	0.006
Spherical equivalent (mean)	-0.24	.05	-0.03	0.73

PS, pupil size. ΔPS, changes in pupil size.

*Multivariate analysis adjusted for age, baseline pupil size, changes in pupil size during accommodation, spherical equivalent.
